# Multimodal Large Language Models Rate Wetlab Suturing Repeatably but Fail to Detect Training-Related Improvement: A Pilot Study With Expert Assessment

**DOI:** 10.7759/cureus.117189

**Published:** 2026-09-30

**Authors:** Jameel Mushtaq, Zaid Alsafi, Robert Brady

**Affiliations:** 1 Ophthalmology, Moorfields Eye Hospital NHS Foundation Trust, London, GBR; 2 Ophthalmology, Western Eye Hospital - Imperial College Healthcare NHS Trust, London, GBR

**Keywords:** multimodal large language models, oculoplastic surgery, ophthalmic simulation training, surgical skills assessment, wetlab training

## Abstract

Background

Objective assessment of technical skill remains a bottleneck in surgical training. Expert rating is valid but consumes scarce faculty time and is susceptible to systematic rater effects, while existing automated methods rely on bespoke computer-vision models that are inaccessible to most educators. General-purpose multimodal large language models can score an image against a written rubric with no task-specific training, but their ability to judge surgical technical quality from a still photograph has not been established.

Materials and methods

Five ophthalmology residents (ST3 to ST6) attending a face-to-face oculoplastic wetlab course each closed a standardised 10 cm linear wound in ex vivo porcine tissue with a running continuous 5-0 polypropylene suture, once before and once after the course, within a fixed 10-minute limit. The 10 resulting closures were photographed under standardised conditions and cropped to the wound. A senior oculoplastic fellow, blinded to delegate identity and to pre- versus post-course status, scored every image on an eight-item anchored rubric (1 to 5 per item). The same images and the same locked rubric prompt were submitted to three multimodal large language models (Claude Opus 4.5, GPT-5.5, and Gemini 3.1 Flash-Lite), three times each at temperature 0, yielding 90 model ratings and 720 item scores from the same 10 photographs. The independent unit of analysis was the delegate (n = 5) for change and the image (n = 10) for agreement. The primary outcome was the pre- to post-course change in a seven-item composite score; secondary outcomes were agreement with the expert, repeatability across identical repeat calls, and completeness of model output.

Results

The expert recorded improvement in all five delegates (composite 2.90 ± 0.65 to 3.71 ± 0.49; change +0.82). Only Gemini 3.1 Flash-Lite reproduced a comparable effect (+0.77); Claude Opus 4.5 (+0.21) and GPT-5.5 (+0.19) did not. Claude Opus 4.5 assigned the scale midpoint of 3 to 72.3% of all item ratings and GPT-5.5 to 68.0%, compared with 39.2% for the expert; no model awarded a score of 5 anywhere in the 720 item scores, and all models rated systematically below the expert (mean item difference −0.45 to −0.73). Rank agreement with the expert for overall closure quality was moderate (Spearman rho 0.65 to 0.79), but item-level agreement was poor (exact agreement 33% to 42%; quadratic weighted kappa 0.20 to 0.24). Repeatability was high, with identical scores across all three repeat calls in 100% (Gemini 3.1 Flash-Lite), 91% (Claude Opus 4.5), and 70% (GPT-5.5) of image-item pairs. Knot security accounted for almost every rating returned as non-assessable.

Conclusions

Contemporary multimodal large language models rated wetlab suturing photographs highly repeatably but with severe central-tendency compression and systematic severity, and two of three failed to detect an improvement that both an expert and one model identified. Repeatability should not be mistaken for validity. With single-image rubric prompting, these models are not yet suitable for summative assessment of surgical technical skill, and methods that widen their effective scoring range are required before formative use can be recommended. These pilot findings, from five delegates and 10 images, are hypothesis-generating.

## Introduction

The reliable assessment of surgical technical skill remains one of the least solved problems in postgraduate surgical education. Structured instruments such as the Objective Structured Assessment of Technical Skills established that expert observers can rate performance reproducibly when rubrics carry explicit anchors and raters are trained [1], and procedure-specific rubrics have since been developed across specialties for the same purpose, including internationally agreed, behaviourally anchored rubrics for ophthalmic procedures [2,3]. Expert rating nevertheless carries two practical costs. It consumes the time of the surgeons who are scarcest, and it is susceptible to systematic rater effects, including leniency, severity, and central tendency, which account for a material share of score variance and require explicit statistical correction to remove [4].

Simulation and wetlab training are now embedded in ophthalmic curricula, and their value is supported by evidence of transfer to the operating room: proficiency-based virtual reality cataract training improves subsequent real operative performance [5], and wetlab teaching more broadly has a consistent role in ophthalmic education [6]. Ex vivo porcine tissue is the standard substrate for oculoplastic wetlab work and has been shown to be a usable training model for periocular procedures [7]. Assessment on such courses, however, is usually informal. Faculty observe, comment, and move on; systematic scoring of every delegate's work at every timepoint is rarely feasible, and course-level evidence of skill acquisition is correspondingly thin.

Automated assessment has been pursued as a solution for over a decade. Video-based deep learning and instrument kinematics can classify skill level with useful accuracy in laparoscopic and robotic settings [8,9], and a recent systematic review confirms both the growth of the field and its persistent problems of methodological heterogeneity, limited transparency, and sparse validity evidence [10]. These approaches share a practical barrier: each requires a bespoke annotated dataset, engineering expertise, and often an instrumented environment, none of which is available to the average course organiser.

Multimodal large language models offer a different route. They are general-purpose, require no task-specific training data, and accept an image together with a written rubric as their entire specification. Evidence on their visual competence in medicine is, however, mixed. A leading chatbot answered image-based ophthalmic questions substantially less accurately than text-based questions [11], and GPT-4V performed below the threshold required for clinical use when detecting findings on chest radiographs [12]. Closest to the present work, two general-purpose vision-language models applied to laparoscopic operative video identified instruments and classified procedures reliably but graded pathology poorly, although in-context learning improved several tasks [13]. Earlier text-only evaluation in ophthalmology showed both genuine capability and characteristic failure modes [14]. As graders in medical education, large language models have shown reasonable agreement with human markers on short-answer text [15] but poor calibration and inconsistent behaviour when marking high-stakes multimodal examinations [16].

What has not been tested is whether such a model can score a still photograph of a completed suture line against an anchored rubric and, more importantly for education, whether it can detect the improvement that a training course produces. We therefore compared three contemporary frontier models with an expert reference on paired pre- and post-course photographs from an ophthalmic surgical wetlab. The primary objective was to determine whether model ratings are sensitive to a known training effect, measured as the pre- to post-course change in the seven-item composite rubric score within each delegate and compared against the change recorded by the expert. Three secondary objectives were addressed: to quantify agreement between each model and the expert, measured by rank correlation across images, by exact, within-one, and chance-corrected agreement at item level, and by mean difference with limits of agreement; to quantify the repeatability of each model, measured as the proportion of identical scores across three repeated calls at a sampling temperature of zero; and to describe the completeness of model output, measured as the proportion of rubric items returned as non-assessable.

## Materials and methods

Setting and participants

This pilot study used images collected during the Moorfields Basic and Advanced Wetlab Course, a face-to-face oculoplastic surgical skills course held at Moorfields Eye Hospital, London, United Kingdom, in April 2026. Five ophthalmology residents at ST3 to ST6 level, with varying prior surgical experience, took part. These five were all of the delegates attending the course; no sampling or selection was applied, and every delegate completed both the pre-course and the post-course assessment task, so the cohort is complete rather than a selected subset. Sample size was determined by course capacity rather than by a formal power calculation, and the study was designed as a pilot.

The assessment task

Immediately before and immediately after the taught programme, each delegate closed a standardised 10 cm linear wound created in ex vivo porcine tissue using a running continuous suture. Closure was performed with 5-0 polypropylene. Delegates chose freely between Barraquer and Castroviejo needle holders, between St Martin's, Jayle's, and Lister forceps, and used straight scissors. A fixed 10-minute limit applied to both attempts for every delegate. Closures that remained incomplete at 10 minutes were photographed as they stood, with no extension of time, so that speed and quality were both reflected in the final image.

Course content

The course comprised a didactic component covering instruments, sutures, tissue handling, and methods of closure, and a practical component on porcine tissue covering wedge resection, laceration repair, tarsorrhaphy, and lateral canthotomy and cantholysis. Senior delegates additionally covered Hughes flap reconstruction and evisceration.

Image acquisition and handling

Photographs were taken with a single smartphone camera (iPhone 13 Pro; Apple Inc., Cupertino, CA, USA) under standardised lighting, from a standardised distance, with a ruler in the field of view. Each image was cropped to the sutured wound alone before assessment. Images were labelled with a delegate code (T01 to T05) and a timepoint for analysis only; for rating, they were presented under anonymised identifiers, in randomised order, blinded to delegate identity and to pre- versus post-course status, so that paired closures from the same delegate were not viewed consecutively. Ten images were available for analysis: one pre-course and one post-course closure for each of the five delegates.

The rating rubric

A purpose-built rubric of eight items was used (Table 1). Seven items addressed specific technical features of a running closure: wound apposition, suture tension, bite symmetry, bite spacing regularity, suture line straightness, tissue eversion, and knot security. Each was scored from 1 to 5 against written descriptors defined at every level. The eighth item, overall closure quality, was a holistic judgement scored on the same scale; raters were explicitly instructed to score it last and not to compute it as a sum or mean of the preceding items. Any item that could not be judged from the photograph could be returned as non-assessable.

Expert reference rating

A senior oculoplastic fellow, who was also faculty on the course, scored all 10 images against the rubric using an electronic form, blinded to delegate identity and to timepoint, with the images presented in randomised order. The rater had co-developed the rubric and was therefore closely familiar with its anchored descriptors; no separate rater-calibration exercise or pilot scoring round was undertaken before the study images were scored. Only one expert rater was available for this pilot. Inter-rater reliability could therefore not be estimated, and the reference standard is not independent of the instrument, since the rater helped construct the rubric against which the models were also scored. The expert scores are accordingly treated throughout as a single reference standard rather than as a criterion of demonstrated reliability, and the consequences are considered in the Limitations.

Model rating

Three multimodal large language models were evaluated: claude-opus-4-5 (Anthropic, San Francisco, CA, USA), gpt-5.5 (OpenAI, San Francisco, CA, USA), and gemini-3.1-flash-lite (Google, Mountain View, CA, USA). All were accessed through their respective application programming interfaces in May 2026 (Table 2). Each image was submitted independently in a single-turn request with identical system and user prompts across all three models. The prompt reproduced the full rubric verbatim, instructed the model to score only the closure visible in the image, prohibited comparison with any other closure and any inference of pre- or post-course status, and required a structured response in JSON containing a score and a one-sentence justification for each item, together with a note on image quality. Sampling temperature was set to 0 and the response limit to 1,500 tokens. Each image was rated three times by each model, giving 90 model ratings and 720 item scores in total. The prompt was locked before the first call and was not modified at any point. No exemplar images, few-shot examples, or fine-tuning were used. Apart from sampling temperature and the response limit, all generation parameters were left at provider defaults, no retrieval or tool use was enabled, and every request was stateless, with no conversation history carried between calls, so the order in which images were submitted could not influence any rating. Models were queried through their published alias endpoints rather than through dated snapshot identifiers, so the precise underlying model build cannot be pinned retrospectively; the alias, developer, and access window for each model are given in Table 2, and the analysis code is available from the corresponding author on request.

Unit of analysis

This design yields a large number of scores from a small number of independent observations, and the two must be kept distinct. The independent unit is the delegate (n = 5) for every analysis of pre- to post-course change and the image (n = 10) for every analysis of agreement with the expert. The 720 item scores do not constitute 720 independent observations: they arise from the same 10 photographs, each scored on eight rubric items that are not independent of one another, by each of three models, on three repeated calls. Repeated calls were therefore averaged to a single value per image and item before any analysis of change or agreement and were used in their own right only to quantify repeatability. Item-level agreement statistics are reported as descriptive summaries pooled across rubric items and are not treated as independent comparisons or subjected to significance testing. No hierarchical or mixed-effects model was fitted because the number of independent units is far too small to support one.

Statistical analysis

For each image and item, the model score was taken as the mean of the three repeat calls. A composite score was calculated as the mean of items 1 to 7, excluding items returned as non-assessable; the holistic item was analysed separately. Pre- to post-course change was summarised by the mean change, Cohen's dz, and the Wilcoxon signed-rank test. With five paired observations, the smallest attainable two-sided p-value is 0.0625, so no comparison in this study can reach conventional statistical significance; tests are reported for completeness, and interpretation rests on effect size and direction of change. Agreement with the expert was assessed by Spearman rank correlation across the 10 images, by exact and within-one agreement and quadratic weighted kappa at item level using the modal model score, and by mean difference with 95% limits of agreement [17]. Repeatability was quantified as the proportion of image-item pairs for which all three repeat calls returned an identical result, counting an item consistently returned as non-assessable as identical, and as the mean within-image standard deviation across repeats of the numeric scores. Analyses were performed in Python 3 using pandas and SciPy. Given five delegates and 10 images, all estimates carry wide uncertainty, no adjustment was made for multiple comparisons, and the findings are hypothesis-generating rather than confirmatory.

Ethical considerations

Ex vivo porcine tissue was obtained from a commercial supplier as a food-industry by-product. No animals were killed for the purposes of this study, and no live animal work was undertaken. Delegates consented to the use of images of their work; the images contained no identifiable information. The study was an educational evaluation of an established training course, and formal research ethics committee approval was not required.

**Table 1:** The eight-item suture closure rating rubric Each item was scored from 1 to 5 against written descriptors defined at every level; the descriptors for scores 2 to 4 are omitted here for brevity. Item 8 was a holistic judgement, scored last and not derived from items 1 to 7. Any item that could not be judged from the photograph could be returned as non-assessable

Item	Score of 1 (lowest)	Score of 5 (highest)
1. Wound apposition	Edges gaping or overlapping along most of the wound; clear dehiscence	Edges precisely apposed end to end with no visible gap or step-off
2. Suture tension	Severely over-tightened with cheese-wiring or blanching, or markedly loose	Uniform, appropriate tension; tissue approximated without distortion
3. Bite symmetry	Bites grossly asymmetric throughout; edges ride at different levels	Each bite mirrors its opposite; edges sit at the same level throughout
4. Bite spacing regularity	Highly irregular; bites bunched in places and widely separated in others	Bites evenly and consistently spaced along the entire wound
5. Suture line straightness	Suture line wanders, zig-zags or crosses the wound	Suture line runs cleanly along the wound axis throughout
6. Tissue eversion	Frank inversion of edges along most of the wound	Appropriate, uniform eversion of wound edges along the full length
7. Knot security	Knot absent, unravelled or clearly insecure at one or both ends	Both knots flat, square and secure, with appropriately trimmed tails
8. Overall closure quality	Closure would require complete revision	Excellent closure; would not require revision or improvement

**Table 2:** Models evaluated and rating configuration All models received identical system and user prompts, were queried through their respective application programming interfaces at a sampling temperature of 0 with a 1,500-token response limit, and returned structured JSON containing a score and a one-sentence justification for each of the eight rubric items. No exemplar images, few-shot examples or fine-tuning were used.

Model	Developer	Access date	Repeat calls per image	Item scores contributed
claude-opus-4-5 (Claude Opus 4.5)	Anthropic	May 2026	3	240
gpt-5.5 (GPT-5.5)	OpenAI	May 2026	3	240
gemini-3.1-flash-lite (Gemini 3.1 Flash-Lite)	Google	May 2026	3	240

## Results

Data completeness

Every one of the 90 model calls returned a valid structured response; there were no application programming interface failures and no parsing errors. Ratings returned as non-assessable were uncommon: 3 of 240 item scores (1.2%) for Gemini 3.1 Flash-Lite, 9 of 240 (3.8%) for Claude Opus 4.5, and 15 of 240 (6.2%) for GPT-5.5. Knot security accounted for all non-assessable ratings from Claude Opus 4.5 and Gemini 3.1 Flash-Lite and for 12 of the 15 from GPT-5.5, the remaining three being tissue eversion. The expert returned one non-assessable rating of 80, also for knot security. All three models judged every image to be assessable overall. As set out in Methods, these 720 item scores derive from 10 photographs and are not independent observations; all comparisons below rest on five delegates and 10 images.

Expert-rated training effect

The expert recorded a clear improvement across the course. The composite score rose from 2.90 ± 0.65 before the course to 3.71 ± 0.49 afterwards, a mean change of +0.82 (Cohen's dz 2.37; Wilcoxon p = 0.063), with improvement in all five delegates. Overall closure quality rose from a mean of 3.00 to 4.00, improving in four of five delegates and remaining unchanged in one. The largest item-level gains were in suture tension (+1.20), bite spacing regularity (+1.00), and knot security (+1.00).

Distribution of scores awarded

The expert used the range 2 to 5, with a mean item score of 3.34 ± 0.83; 39.2% of ratings were 3, 38.0% were 4, and 6.3% were 5 (Figure 1). The models were markedly more compressed. Claude Opus 4.5 assigned a score of 3 to 72.3% of all item ratings and GPT-5.5 to 68.0%. No model awarded a score of 5 at any point across 720 item scores, and only a single rating of 1 was recorded, by GPT-5.5. Gemini 3.1 Flash-Lite was the most dispersed of the models, though its dispersion sat lower on the scale, with 49.4% of ratings at 2, 40.5% at 3, and 10.1% at 4. Mean item scores were 2.88 ± 0.51 (Claude Opus 4.5), 2.80 ± 0.54 (GPT-5.5), and 2.61 ± 0.67 (Gemini 3.1 Flash-Lite), all below the expert mean.

**Figure 1:**
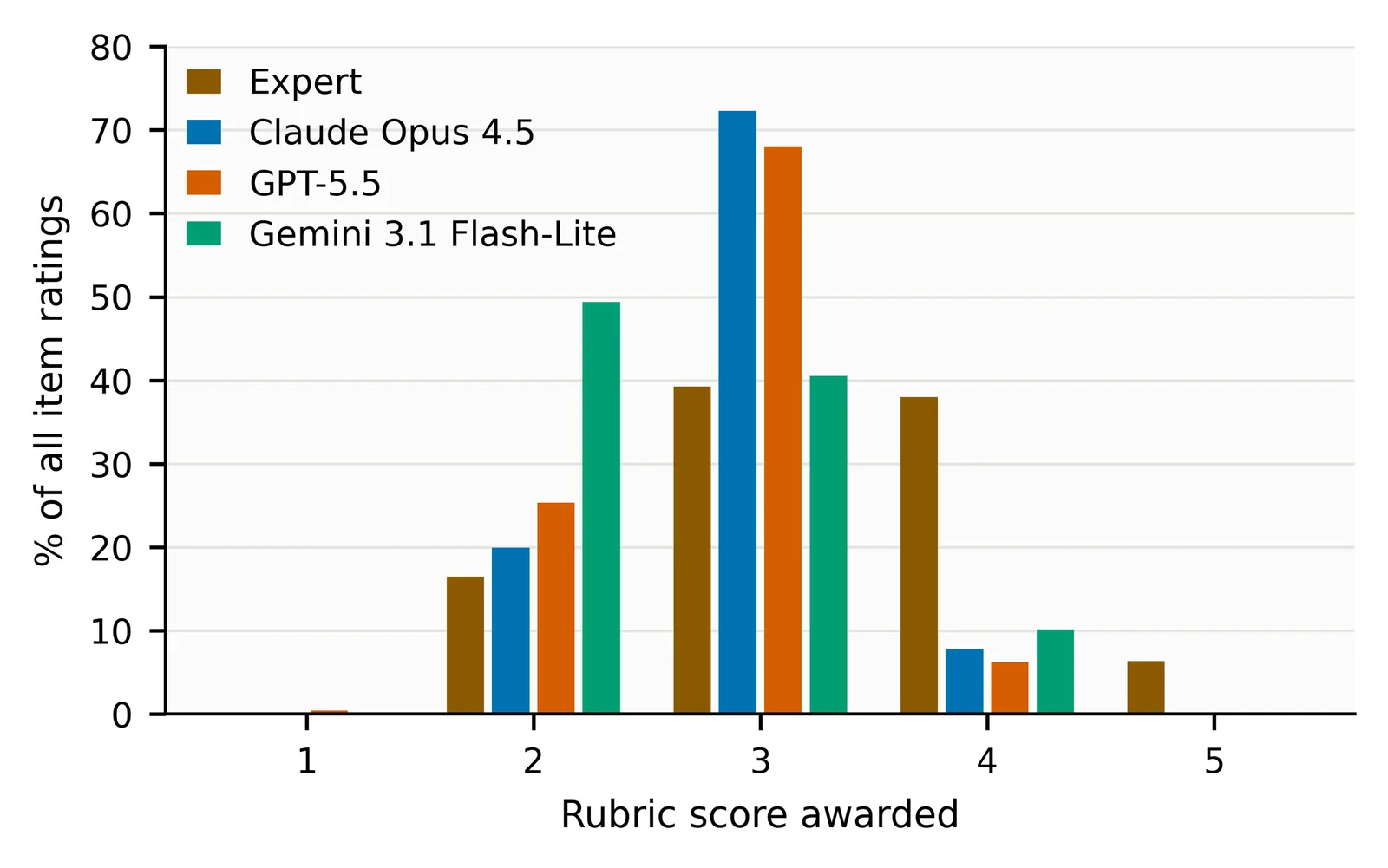
Distribution of scores awarded across all rubric items Expert n = 79 item ratings; each model n = 240 item ratings (8 items x 10 images x 3 repeat calls). Ratings returned as non-assessable are excluded. No model awarded a score of 5 at any point.

Detection of pre- to post-course change

Against the expert's improvement (Figure 2A), only one model reproduced a comparable training effect (Table 3). Gemini 3.1 Flash-Lite recorded a composite change of +0.77 (dz 1.67), with improvement in all five delegates, and its holistic score rose from 2.00 to 3.00 (Figure 2D). GPT-5.5 recorded higher post-course composite scores in all five delegates, but the mean change was only +0.19 (dz 0.96), roughly one quarter of the change the expert recorded, and its holistic score improved in only one of five delegates (mean change +0.07) (Figure 2C). Claude Opus 4.5 recorded a composite change of +0.21 (dz 0.52), with improvement in three of five delegates on the composite and two of five on the holistic item (mean change +0.07) (Figure 2B). The discordance is illustrated by the delegate whom the expert scored highest at both timepoints, whose composite rose from 3.86 to 4.57 in the expert's hands but moved from 3.14 to 3.00 for Claude Opus 4.5 and from 3.00 to 3.05 for GPT-5.5.

**Figure 2:**
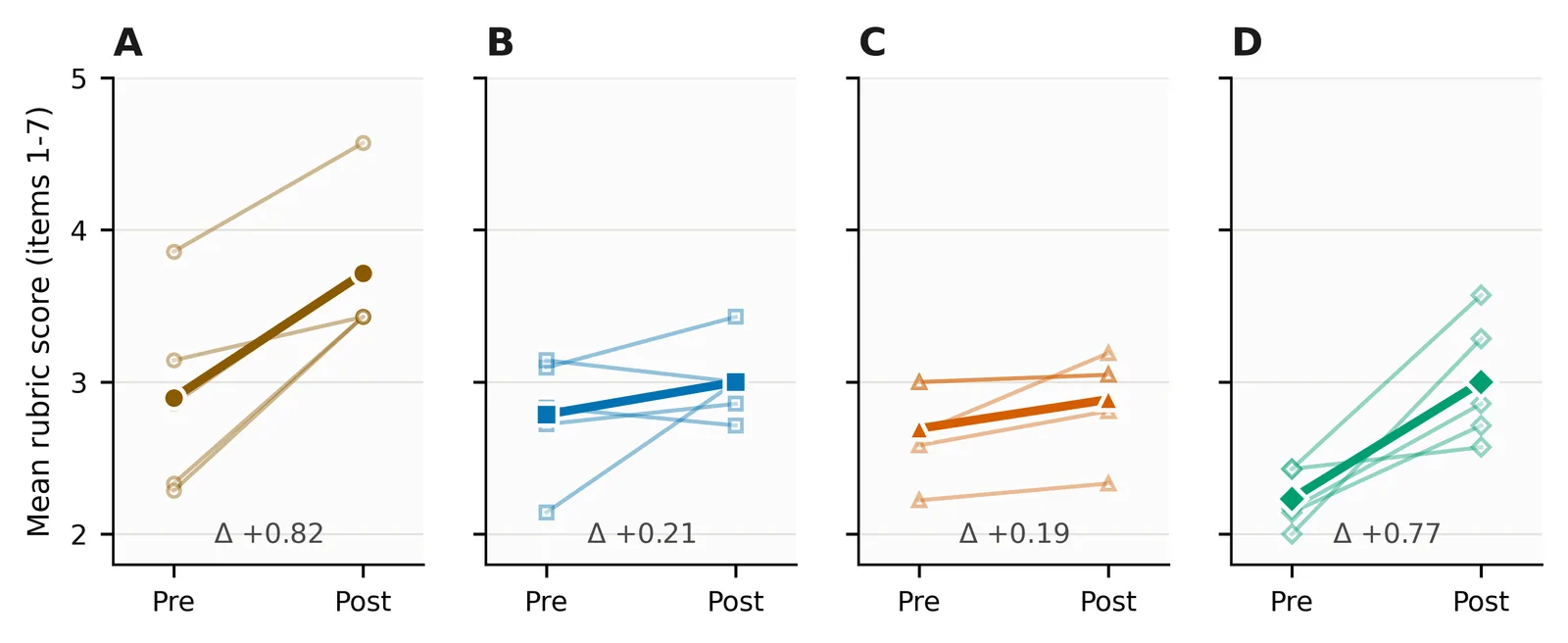
Pre- to post-course change in mean rubric score, by rater (A) Expert rater. (B) Claude Opus 4.5. (C) GPT-5.5. (D) Gemini 3.1 Flash-Lite. In every panel the composite score is the mean of rubric items 1 to 7; thin lines show individual delegates (n = 5) and the thick line shows the group mean, with the mean pre- to post-course change annotated. Model scores are the mean of three repeat ratings per image. The expert (A) and Gemini 3.1 Flash-Lite (D) show comparable improvement, whereas Claude Opus 4.5 (B) and GPT-5.5 (C) are largely flat.

**Table 3:** Pre- and post-course scores by rater Composite score is the mean of rubric items 1 to 7 (scale 1 to 5). Holistic refers to rubric item 8, overall closure quality. dz is Cohen's dz for paired data. With five paired observations the smallest attainable two-sided Wilcoxon p value is 0.063, so no comparison can reach conventional significance; p values are reported for completeness only. SD, standard deviation.

Rater	Composite pre (mean ± SD)	Composite post (mean ± SD)	Change	dz	Wilcoxon p	Delegates improved (composite / holistic)
Expert	2.90 ± 0.65	3.71 ± 0.49	+0.82	2.37	0.063	5/5 / 4/5
Claude Opus 4.5	2.79 ± 0.40	3.00 ± 0.27	+0.21	0.52	0.438	3/5 / 2/5
GPT-5.5	2.69 ± 0.33	2.89 ± 0.34	+0.19	0.96	0.063	5/5 / 1/5
Gemini 3.1 Flash-Lite	2.23 ± 0.19	3.00 ± 0.42	+0.77	1.67	0.063	5/5 / 4/5

Agreement with the expert

Rank agreement for overall closure quality was moderate for all three models: Spearman rho 0.79 (p = 0.007) for GPT-5.5 (Figure 3B), 0.66 (p = 0.038) for Claude Opus 4.5 (Figure 3A), and 0.65 (p = 0.043) for Gemini 3.1 Flash-Lite (Figure 3C) (Table 4). Agreement for the seven-item composite was weaker and did not reach nominal significance for any model (rho 0.45 to 0.58). All three models were systematically severe relative to the expert, with mean item-level differences of −0.45 (Claude Opus 4.5), −0.54 (GPT-5.5), and −0.73 (Gemini 3.1 Flash-Lite); for overall closure quality, the mean differences were −0.73, −0.73, and −1.00, respectively, with wide limits of agreement (for example, −2.30 to +0.83 for Claude Opus 4.5). At item level, exact agreement with the expert was 42% for Claude Opus 4.5, 36% for GPT-5.5, and 33% for Gemini 3.1 Flash-Lite; agreement to within one point was 91%, 90%, and 82%, respectively. Quadratic weighted kappa was 0.20, 0.23, and 0.24, corresponding to slight to fair agreement on conventional thresholds [18]. 

**Figure 3:**
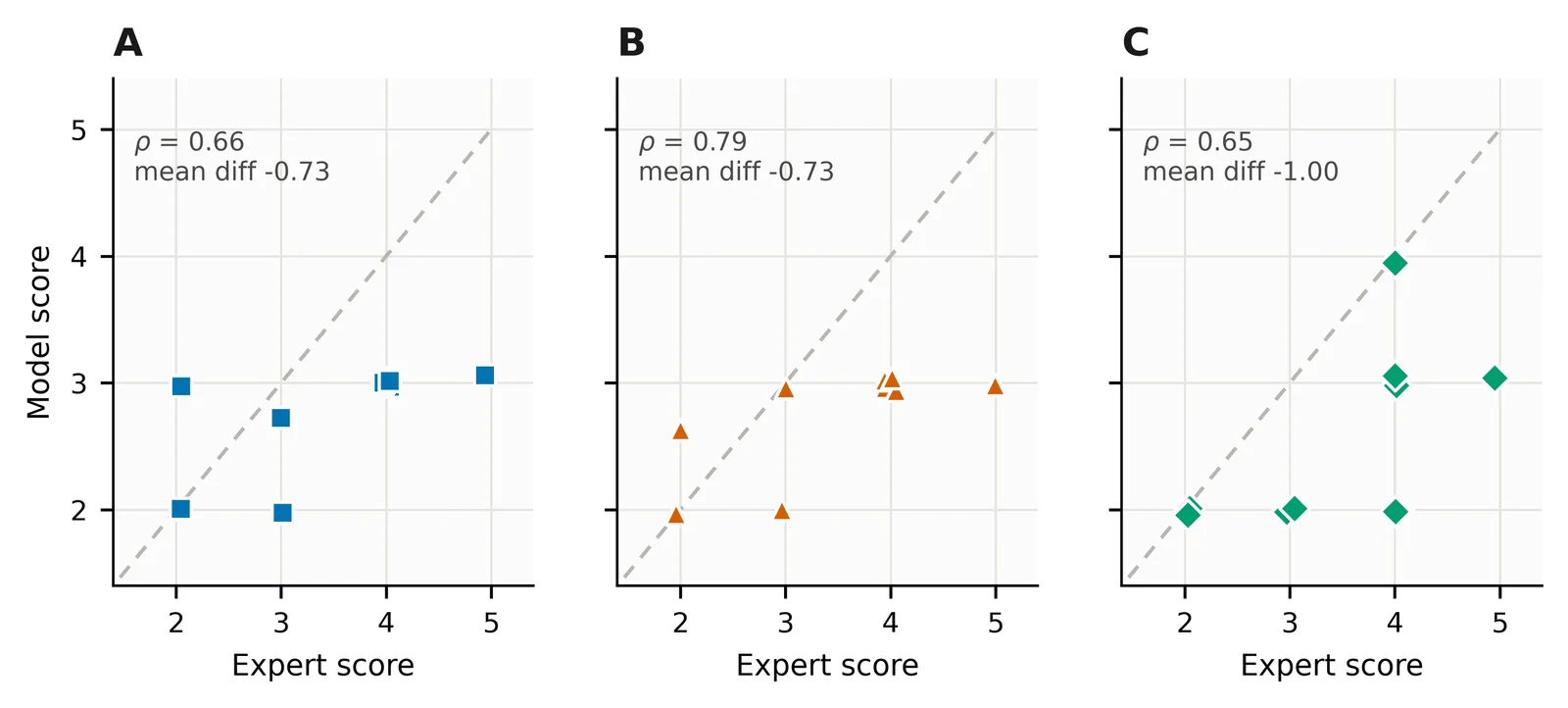
Model versus expert rating of overall closure quality (A) Claude Opus 4.5. (B) GPT-5.5. (C) Gemini 3.1 Flash-Lite. In every panel each point represents one image (n = 10), jittered to separate tied values, and the dashed line indicates perfect agreement; the Spearman correlation with the expert and the mean model-minus-expert difference are annotated. All three models fall predominantly below the line of equality, indicating systematic severity relative to the expert.

**Table 4:** Agreement between each model and the expert rater Correlations are calculated across the 10 images. Item-level agreement statistics use the modal model score across three repeat calls, compared with the expert score, for all eight rubric items. Weighted kappa is quadratic weighted kappa. Negative mean differences indicate that the model scored below the expert.

Model	Spearman rho, holistic (p)	Spearman rho, composite (p)	Exact agreement	Within 1 point	Weighted kappa	Mean difference, item level (holistic, 95% limits of agreement)
Claude Opus 4.5	0.66 (0.038)	0.55 (0.101)	42%	91%	0.20	−0.45 (−0.73; −2.30 to +0.83)
GPT-5.5	0.79 (0.007)	0.58 (0.080)	36%	90%	0.23	−0.54 (−0.73; −2.20 to +0.74)
Gemini 3.1 Flash-Lite	0.65 (0.043)	0.45 (0.193)	33%	82%	0.24	−0.73 (−1.00; −2.60 to +0.60)

Repeatability and between-model agreement

Repeatability across identical repeat calls at temperature 0 was high (Table 5). Gemini 3.1 Flash-Lite returned an identical result on all three calls for every one of its 80 image-item pairs, Claude Opus 4.5 for 91%, and GPT-5.5 for 70%; mean within-image standard deviations of the numeric scores were 0.00, 0.05, and 0.16, respectively. The least repeatable item was knot security for GPT-5.5, identical across all three calls in only four of 10 images; for Claude Opus 4.5, the least repeatable items were bite spacing regularity and tissue eversion, each identical in eight of 10 images. Agreement between models was moderate and no greater than their agreement with the expert: pairwise Spearman correlations for overall closure quality ranged from 0.51 to 0.55, and for the composite from 0.43 (Claude Opus 4.5 with Gemini 3.1 Flash-Lite) to 0.70 (Claude Opus 4.5 with GPT-5.5). The mean standard deviation between the three models' holistic scores for a given image was 0.32 points. 

**Table 5:** Repeatability across identical repeat calls and completeness of rating All calls were made at a sampling temperature of 0. Repeatability is the proportion of the 80 image-item pairs per model for which all three repeat calls returned an identical result, counting an item consistently returned as non-assessable as identical. Knot security accounted for all non-assessable ratings from Claude Opus 4.5 and Gemini 3.1 Flash-Lite and for 12 of 15 from GPT-5.5. SD, standard deviation.

Model	Identical across all 3 calls	Mean within-image SD	Non-assessable ratings	Least repeatable item
Claude Opus 4.5	91% (73/80)	0.05	9/240 (3.8%)	Bite spacing, tissue eversion
GPT-5.5	70% (56/80)	0.16	15/240 (6.2%)	Knot security
Gemini 3.1 Flash-Lite	100% (80/80)	0.00	3/240 (1.2%)	None; all identical

## Discussion

Three contemporary multimodal large language models, given the same anchored rubric and the same photographs as an expert rater, produced ratings that were highly repeatable and moderately rank-correlated with the expert on holistic quality, yet two of the three failed to detect the pre- to post-course improvement that the expert recorded in every delegate. For course evaluation or formative feedback, that failure would be disqualifying. The quantity of educational interest is change, not absolute level, and a rating instrument that cannot register change cannot support either purpose.

The mechanism is visible in the score distributions. When approximately 70% of ratings land on the midpoint of a five-point scale, there is very little room left in which to express improvement. The expert used the upper half of the scale freely, with 44% of ratings at 4 or 5, whereas no model awarded a single 5 across 720 item scores. Two consequences follow: systematic severity, with models rating 0.45 to 0.73 points below the expert on average, and range restriction, with model standard deviations of 0.51 to 0.67 against the expert's 0.83. The models were not so much disagreeing with the expert about which closures were better as declining to commit to how much better.

A plausible explanation is that these models are tuned toward hedged and defensible outputs and that a verbal rubric is a weak calibrator in the absence of comparative context. Each image was scored in isolation, with no exemplars of what a 1 or a 5 looks like in this tissue, at this magnification, for this task. A human rater calibrates against years of accumulated visual reference; a model prompted cold has only the text. This interpretation is consistent with reports that large language models can rank medical answers acceptably while calibrating absolute marks poorly, particularly on multimodal material [15,16].

Our results also illustrate that repeatability is not validity. Gemini 3.1 Flash-Lite, the smallest and least expensive model tested, was simultaneously the most deterministic, returning an identical result on every one of its 80 repeated image-item ratings, and the only model to track the training effect. GPT-5.5 was the least repeatable of the three yet produced the strongest rank correlation with the expert on holistic quality. Neither reliability metric predicted the educationally relevant behaviour. Evaluations of models as assessors that report only inter-run consistency or correlation with a reference will therefore overstate readiness, and we would argue that sensitivity to a known change should be reported as a primary outcome in this literature.

That the model which discriminated best was also the smallest is an observation rather than a finding. With three models, 10 images, and one expert, it cannot be attributed to scale, tuning, or chance. It does, however, caution against the assumption that the largest available model will make the best assessor, and it argues for empirical testing of any model intended for this role.

Knot security was the item most frequently returned as non-assessable by every rater, the expert included. A single cropped photograph of a completed running closure frequently does not display both anchoring knots. This is a limitation of the imaging protocol rather than of the models, and future protocols should either acquire dedicated views of each knot or omit the item from photograph-based rating.

Several routes might close the gap we observed. Few-shot anchoring, in which reference photographs at defined score levels accompany the rubric, addresses the calibration deficit most directly, and in-context learning of this kind has already been shown to improve general-purpose model performance on surgical video tasks [13]. A related but distinct possibility is contextual calibration within a persistent assessment session: rather than presenting fixed exemplars alongside every request, a model would score a series of closures within one continuous conversation, accumulating exposure to the range of work actually in front of it and anchoring its use of the scale against that range rather than against verbal descriptors alone. This is in-context learning without retraining, and it is exactly what our stateless design excludes by construction. Comparative rather than absolute judgement, presenting the paired pre- and post-course images and asking which closure is better, converts the task into a discrimination that models may perform more reliably and maps naturally onto course evaluation; comparative judgement is an established assessment method in medical education, achieving reliability comparable to rubric scoring for the same time investment [19]. Explicit distributional instruction, or post hoc calibration of model output onto the expert scale, may partially correct central tendency without changing the model. Fine-tuning on expert-rated images would likely be most effective but sacrifices the generality that makes the approach attractive to educators in the first place. Until at least one of these is shown to work, rating of suture photographs by multimodal large language models should not be used summatively. Any formative use should rest on the models' written justifications, which were specific and rubric-anchored even where the numbers were not discriminating, rather than on the scores themselves.

Limitations

This study has substantial limitations, and they bear directly on how far its findings can be pressed. It involved five delegates and 10 images, so every estimate carries wide uncertainty, and the design is powered only to detect large effects; with five paired observations, no test could have reached conventional significance. The volume of individual scores should not obscure this. The 720 item scores arise from 10 photographs rated repeatedly on correlated items and are not independent observations; all inference here rests on five delegates and 10 images, which is why repeated calls were averaged before analysis and item-level agreement is reported descriptively rather than tested. There was also no control group, so the improvement the expert recorded cannot be attributed to the course with confidence. Delegates performed the same task twice, and practice or retest effects arising from simple repetition of an unfamiliar task cannot be separated from the effect of teaching; a design with an untaught comparison group or with a second baseline attempt would be needed to do so.

Limitations also attach to the measurement. Only one expert rater was available, that rater was also faculty on the course, and the rater had co-developed the rubric, so the intraclass correlation coefficient and related measures of rater agreement could not be estimated [20], the reference standard is not independent of the instrument, and expectation effects cannot be excluded despite blinding to delegate and timepoint and randomisation of presentation order. No separate rater-calibration exercise was undertaken. Models were queried through alias endpoints rather than dated snapshots, so the precise model builds cannot be pinned retrospectively and identical reproduction of these outputs cannot be guaranteed; more broadly, hosted model versions are a moving target, and these findings describe three specific models as accessed in May 2026.

A further and important boundary on generalisability follows from the design itself. Every image was assessed in a single-turn, stateless request, so what we report is the zero-shot performance of these models on one photograph at a time. That isolation is deliberate, since it prevents earlier images and earlier ratings from influencing later ones and is what allows each score to be treated as independent of the others; however, it also means these findings describe only stateless, zero-shot assessment and say nothing about how the same models would behave within a persistent conversational session, where exposure to previously assessed closures or to reference exemplars could calibrate their use of the scale through in-context learning rather than retraining. The central-tendency compression we observed may therefore be in part an artefact of assessing each closure in isolation and should not be assumed to persist under contextual calibration. The work was also conducted at a single institution using a single tissue model and a single task, and a still photograph necessarily discards the process information, including instrument handling, economy of motion, and tissue trauma, that expert raters use in live assessment, so the construct measured here is the quality of the finished closure rather than surgical technique as a whole. The prompt was deliberately locked before the first call to prevent post hoc optimisation, which is methodologically desirable but means we tested one prompting strategy rather than the best achievable one. Finally, the composite treats ordinal items as equally weighted and interval-scaled, a common simplification that should be borne in mind when interpreting mean changes.

## Conclusions

In this pilot comparison, three contemporary multimodal large language models scored photographs of wetlab suturing with high internal repeatability but with marked central-tendency compression and systematic severity relative to an expert rater. Only one of the three models reproduced the pre- to post-course improvement that the expert recorded in every delegate, and item-level agreement with the expert was, at best, fair. Repeatability and moderate rank correlation therefore appear to be poor proxies for the property that matters most in surgical education, namely sensitivity to change in performance. These observations derive from five delegates and 10 images assessed against a single expert reference and should be read as hypothesis-generating rather than as a definitive account of what these models can do.

These results do not argue against the approach so much as against deploying it in its simplest form. Rubric text alone appears insufficient to calibrate a general-purpose model onto a five-point surgical rating scale, and the obvious next steps are all readily testable: visual exemplars at defined score levels, contextual calibration within a persistent assessment session rather than the stateless, single-image requests used here, paired comparative judgement in place of absolute scoring, and validation against several independent expert raters in a larger cohort. On the evidence of this pilot, multimodal large language models are not yet ready for the summative assessment of surgical technical skill, and educators evaluating them should treat sensitivity to a known training effect, rather than agreement or consistency alone, as the primary test of usefulness.
